# Synthetic sugar for sustainable power?

**DOI:** 10.3389/fchem.2014.00013

**Published:** 2014-03-25

**Authors:** Khaled Moustafa

**Affiliations:** Institut Mondor de la Recherche Biomédicale - Institut National de la Santé et de la Recherche MédicaleCréteil, France

**Keywords:** artificial photosynthesis, bioethanol, biofuels, carbon dioxide recycling, green environment, sustainable bioenergy, synthetic sugar

Energy is a major challenge for the future with important consequences on the economic and political stability, food security, water supply, energy independence, pollution, and global ecosystem. Owing to increased industrial activities and growing population, the reserve of oil and other fossil resources will become, at best, rare, and costly for extraction and commercialization.

For these reasons, sustainable energy is on the top interests of scientific and political programs in many countries where economic and industrial activities are main contributors of the emission of greenhouse gases. High accumulation of these gases is predictable to affect agriculture and to sharpen climate change in the next decades. To alleviate these effects, scientists and stakeholders need to addresses all potential hypotheses and approaches to develop sustainable energy systems that minimize the pollution and the negative effects of carbon dioxide (CO_2_) on the biosphere. Toward this objective, different approaches are currently under investigation based on various platforms such as water, microorganisms, plants and photovoltaic devices. Splitting of water, for example, is used to generate gaseous hydrogen using molecular catalysts such as molybdenum-oxo complex (Karunadasa et al., 2010), copper oxide photocathode coated with an amorphous molybdenum sulphide (Morales-Guio et al., 2014) or Silicon/Hematite core/shell nanowire array covered with gold nanoparticles (Wang et al., 2014). Various microorganism genera are also investigated such as microalgae (Gong and Jiang, 2011), bacteria (Kalscheuer et al., 2006), cyanobacteria (Quintana et al., 2011) and yeast (Buijs et al., 2013). Other options include genetically modified plants such as sugarcane (Arruda, 2012), maize (Torney et al., 2007), sunflower and soybean (Dizge et al., 2009). Each of these platforms has its own pros and cons. The production of biohydrogen for example encounters two major challenges relating to high production cost and low-yield (Gupta et al., 2013). Important sustainability issues also obstruct the production of biofuel from algae and plant platforms at industrial viable scales, admitting that a severe compromise would be accepted for biofuel production from crops to the detriment of food production. Moreover, crops-based biofuels can provide only a small portion of the huge amounts of fuels needed annually, estimated at the equivalent of 31 billion barrels of crude oil (Rhodes, 2009). Plant-based biofuels also require precious sources (water and arable lands) that are increasingly scarce, and it is much wiser to save them for more vital needs than energy. Algae, on the other hand, have greater potential than plants to be good alternative resources for renewable energy. However, algae require substantial amount of water and fertilizer resources for scalable production of fuels (Edmundson and Wilkie, 2013). Algal fuels also cannot be produced in large volumes in an environmentally sustainable manner, although crude oil has been produced in various experimental scales (Chisti, 2013). Moreover, there is a permanent need to fully recycle the nitrogen and phosphorous nutrients that are necessary for the sustainable algal biofuel system.

**Figure 1:**
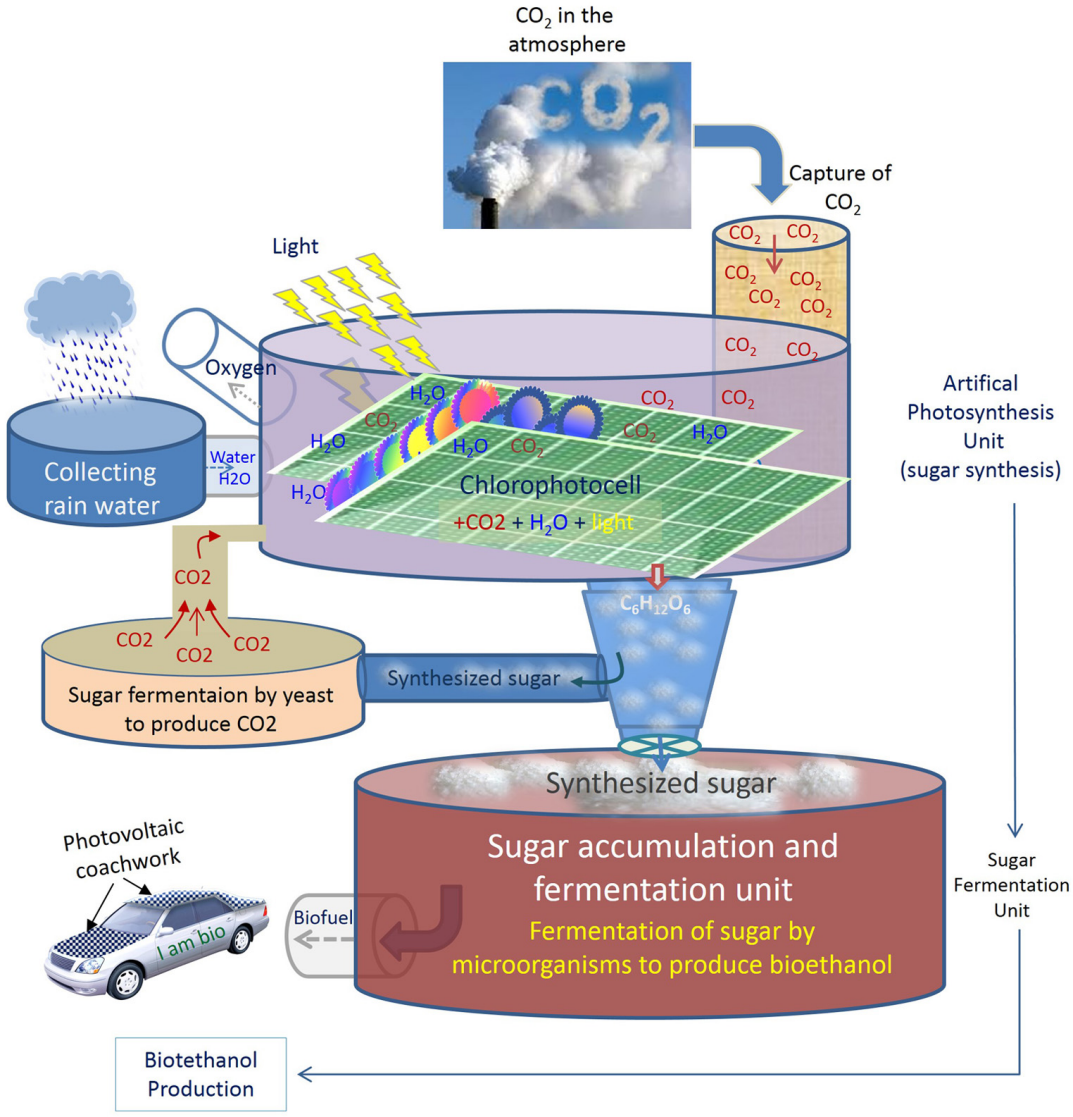
**General scheme of an artificial biosynthesis for the production of bioethanol from synthesized sugar**. To save valuable crops (e.g., maize, beet) and water for more vital applications, an artificial photosynthesis can be used to produce fermentable sugar from CO_2_ and water in a kind of artificial plastids (shown here as Chlorophotocell) as a photo bioreactor for sustainable energy production. An artificial photosynthesis system could be composed of a large unit with artificial plastids to capture solar light and atmospheric CO_2_ and then convert them into sugar by chemical reaction. Produced sugar will be collected in another unit (fermentation unit) where microorganisms can ferment the sugar and produce bioethanol in optimized fermentation conditions. To insure CO_2_ auto-feedback, in case of insufficient atmospheric CO_2_ concentration, a portion of the synthesized sugar can be conducted to another fermentation chamber to produce CO_2_ by yeast and store it to feed the system at the needed moment. Adding natural plant debris as a source of minerals for microorganisms would be necessary to improve the fermentation efficiency. An adapted reservoir can be designed to capture and filter the rain water to supply the requirements of water.

Other alternative energy resources include nuclear and solar-based platforms. Nuclear is a highly risk adventure at local and global level, especially under unpreventable environmental disasters and uncontrollable climate change. The other safer approach is sunlight and its applications with photovoltaic devices. An ideal energy system, however, should offer the possibility to produce sustainable power while reducing CO_2_ emissions at the same time. An enhanced artificial photosynthesis protocol that uses CO_2_, water and sunlight to produce fermentable organic matter (sugar), in the same way that plants do for their own natural photosynthesis, would be an ultimate, or at least a complementary solution to other potential bioenergy alternatives, for both the production of biofuels and the reduction of CO_2_ levels. Figure 1 illustrates a streamlined scheme for a conceptual energy system inspired from photosynthesis process, starting by capturing sunlight and CO_2_ and ending by the synthesis of sugars. The synthetized sugar can then be conducted to a chamber (i.e., fermentation reservoir) where it could be converted to bioethanol by fermentation. Once realized, such an approach would not only offer the advantage to save valuable lands, freshwater and crops, but also to reduce CO_2_ levels and make industrial activities as desirable activities rather than culprit operations, because the CO_2_ emitted will be recycled permanently to feed the artificial system and produce bioethanol. In other term, the more CO_2_ emitted by industrial activities, the more sugar synthesized, and the more bioethanol produced. To enable maximum fixation of CO_2_, such a system is expected to be implemented near industrial factories that emit CO_2_ intensively.

The composition, size, chemical, and physical properties of appropriate devices and materials intended for such a technology need to be scrutinized and custom-made for the specific projected task. This will entail efforts at the interface of multidisciplinary fields including biotechnology, chemistry, physics, engineering and plant biology. Moreover, in an ideal conception, a good sustainable energy platform should combine artificial photosynthesis with photovoltaic devices to take advantages from both settings in all circumstances.

Unlike the natural photosynthesis, whose rate is relatively low and depends on plant species and ambient conditions, the artificial system should enable better control and management of the bioenergy yield. In fact, natural photosynthesis yield is not necessarily low, but just adapted to the needs of photosynthetic organisms. Artificial photosynthesis, on the other hand, is needed to offer more flexibility on the control of the production yield and to satisfy great energy demands. Yet, one of the foremost challenges of sunlight-based technology is what to do when there is little or no sun? To address this intermittent nature of sunlight, an efficient photosynthesis system should mimic plants in their perfect absorption and tackling the light spectrum for their own photosynthesis. From hot and wet to cold and rainy environments, plants capture light efficiently and develop intense vegetation with important organic matter produced (e.g., equatorial and boreal forests). Similarly, an artificial photosynthesis should be optimized to operate in all circumstances of light shifts and intensity. To achieve such an objective, future multidisciplinary advancements and doubled efforts will be necessary to build an effective artificial photosynthesis that could be scalable for industrial production of bioenergy. The conversion of CO_2_→sugar→bioethanol may result in loss of some energy efficiency. To avoid such potential losses and take advantage of a shorter way to convert CO_2_ to fuel, another option would be the conversion of CO_2_ directly to ethanol (United States Patent 8,313,634).

Some other drawbacks may also encounter the outlined approach. Developing innovative technology requires great efforts at basic and applied research until an efficient protocol is reached to reduce CO_2_ to C_6_ sugars. As for any new or unexplored research areas, a substantial investment also will be needed, at least at the first stages before an optimization and automation reduces the cost and augments the sensitivity of the system. The approach will demand great versatilities and sophisticated setting with several phases. For example, to circumvent the issue of CO_2_ dilution in the atmosphere, CO_2_ can be injected into the system after either chemical or, preferably, biological production of CO_2_ by the system itself (e.g., yeast fermentation) (Figure 1). If the CO_2_ concentration is under an optimized threshold, determined for an efficient photosynthesis output, an injection of stored CO_2_ can be programmed to feed the system at the right moment. As such, the system will insure its auto feedback permanently; the fermentation of synthesized sugar by yeast will produce CO_2_, which in turn feeds the photosynthesis system to produce sugar for bioethanol production and so on. A combinatory system can also be imagined in which a starting amount of “natural sugar” might be added sparsely to improve the fermentation quality.

There is no doubt that earth's climate will change inevitably over the next decades due to intense anthropogenic activities. The negative effects of fossil energy combined to unsustainable practices in agricultural and industrial activities will continue to cause damages. To avoid such damages, it is urgent to reduce the emission of greenhouse gases at local and global levels. In the light of the depletion of fossil fuels, considerable investment is mandatory to develop safer and sustainable energy source to avert worst-case scenario. The establishment of new sustainable energies will require biologists, chemist and physicists to narrowly collaborate and examine a wide range of approaches. Artificial photosynthesis to produce synthetic sugar for fermentation and bioethanol production would be a worthy option to focus on. The apparent benefit of such an approach is to reduce CO_2_ levels and to provide renewable clean bioenergy. As supplementary advantages, it is not necessary to use freshwater water nor arable lands, since artificial photosystem could be implemented anywhere. Rain water could also be collected and stored in the same setting to answer the needs of water required for the production of sugars, so an important burden on freshwater can be avoided.

Such an approach holds great potential to be used at least as a complementary approach to the other investigated methods. Further advantages include energy independence and new jobs created as a result of an implementation of new industry and researches for continual optimization and improvement at both basic and applied levels.
